# Impact of breast reconstruction and different surgical approaches after neoadjuvant therapy on the long-term survival of breast cancer patients

**DOI:** 10.1016/j.tranon.2026.102737

**Published:** 2026-03-17

**Authors:** ZhaoQi Qiu, YuFeng Zhang, Ting Shou, YuRong Chen, LieJiong Wang, ZeMing Wang

**Affiliations:** a Department of Oncology Radiotherapy, Zhuji Affiliated Hospital of Wenzhou Medical University, Zhuji, Zhejiang Province, China; b Nursing Group of Zhuji Technical College, Zhuji, Zhejiang Province, China; c Department of Medical Oncology, Zhuji Affiliated Hospital of Wenzhou Medical University, Zhuji, Zhejiang Province, China; d Medical Oncology, Department of Integrative Medicine, Shengzhou People's Hospital (Shengzhou Branch of the First Affiliated Hospital of Zhejiang University School of Medicine, the Shengzhou Hospital of Shaoxing University), Shengzhou, Zhejiang Province, China

**Keywords:** Breast cancer, Neoadjuvant therapy, Breast reconstruction, Nipple-sparing mastectomy, Total mastectomy

## Abstract

**Introduction:**

Large-scale studies on long-term survival after breast reconstruction (BR) following neoadjuvant therapy (NAT) are limited. This study compares long-term survival in breast cancer patients who had BR after NAT—either with nipple-sparing mastectomy (NSM) or total mastectomy (TM)—versus TM alone, to identify subgroups most likely to benefit.

**Methods:**

We analyzed female breast cancer patients who received NAT in the SEER database, stratifying them into three groups: NSM with BR (*n* = 949), TM with BR (*n* = 3554), and TM without BR (*n* = 5465). Overall survival (OS) and breast cancer–specific survival (BCSS) were assessed using Cox proportional hazards models and propensity score matching (PSM).

**Results:**

A total of 9968 patients were included in this study. Compared with TM alone, patients who underwent TM with BR had similar OS (Model 4, Table 3) and BCSS (HR: 0.90–0.96; all *P* > .05; Table 3). However, those who underwent NSM with BR were associated with better OS (HR: 0.29–0.60; all *P* < .05; Table 3) and BCSS (HR:0.31–0.64; all *P* < .05; Table 3). This association remained significant across PSM and multiple association inference models (Table 4) and was more robust in prespecified subgroups—including those aged <65 years and those with earlier-stage disease (Figure 3).

**Conclusions:**

This nationwide cohort study shows that, among patients receiving NAT, NSM with BR is associated with better OS and BCSS than TM alone—whereas TM with BR showed no survival benefit. NSM with BR is a feasible option for selected patients and warrants prospective validation.

## Highlights

• Provides robust long-term survival data from a large-scale study, addressing the impact of Breast reconstruction (BR) on survival outcomes after neoadjuvant therapy (NAT).
• BR after Nipple-sparing mastectomy (NSM) results in better overall survival (OS) and breast cancer-specific survival (BCSS) compared to TM alone. This association remained significant across propensity score matching (PSM) and multiple association inference models (Table 4).
• Specific subgroups, such as younger patients, married patients, those with fewer lymph node metastases, favorable tumor pathology, and timely diagnosis, benefit most from BR after NSM. This suggests personalized treatment strategies for breast cancer patients.

## Introduction

Breast cancer has emerged as a leading cause of cancer-related mortality among women. However, advancements in oncological diagnostics and therapeutics have significantly prolonged patient survival [1]. Consequently, without compromising oncological outcomes, an increasing number of patients are prioritizing quality of life and socio-psychological well-being following breast surgery. Although neoadjuvant therapy (NAT) can enhance the feasibility of breast-conserving surgery (BCS), thereby improving patient satisfaction and reducing psychosocial burden [[2], [3], [4]], many patients still opt for total mastectomy (TM) after NAT due to disease staging and personal preferences. Immediate or delayed breast reconstruction (BR) after TM can partially substitute for BCS, alleviating anxiety about tumor recurrence and enhancing quality of life [5,6].

Nipple-sparing mastectomy (NSM) involves the removal of breast tissue while preserving the nipple-areola complex and overlying skin. Due to its ability to maintain much of the skin envelope and inframammary fold after reconstruction, NSM provides superior aesthetic outcomes and is increasingly preferred by younger breast cancer patients [7]. However, concerns remain regarding the potential increased risk of local recurrence, leading patients with locally advanced breast cancer (LABC) to be hesitant about undergoing NSM [8]. With the growing use of NAT in breast cancer treatment, which significantly improves pathological response rates and mitigates disease severity, NSM combined with BR following NAT is considered a viable option without raising the incidence of postoperative complications [[9], [10], [11], [12], [13]]. As a result, the utilization of NSM and BR after NAT is on the rise [14]. While some studies have confirmed that the oncological outcomes of NSM and BR following NAT are comparable to, or even superior to, those of TM [[15], [16], [17], [18], [19]], there is a paucity of large-sample studies examining long-term survival in the U.S. breast cancer population. Additionally, few studies further investigate which patient populations are most suitable for NSM and BR surgery. Therefore, this study aims to compare the long-term survival outcomes of NSM and BR versus TM in U.S. breast cancer patients following NAT and to identify specific populations that may benefit from NSM and BR surgery.

## Materials and methods

### Study design

To evaluate the potential long-term survival benefits associated with breast reconstruction in this specific patient cohort, we categorized the included population into three groups based on whether they underwent reconstruction and the type of surgical approach. These groups served as the independent variables, while overall survival (OS) and breast cancer-specific survival (BCSS) were the dependent variables. The maximum follow-up period was 131 months, with a median follow-up duration of 31.9 months, and follow-up continued until the end of 2022.

### Study population

**Fig. 1:**
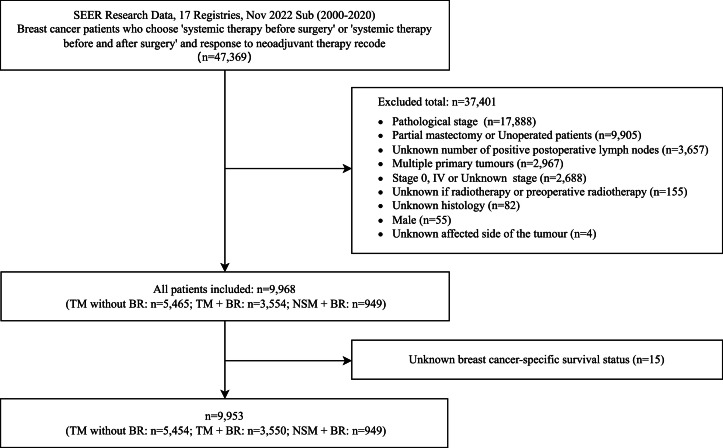
Flow chart of the patient selection process.

We utilized patient population data from the Surveillance, Epidemiology, and End Results (SEER) database [20]. Patients diagnosed with breast cancer (International Classification of Diseases for Oncology, Third Edition ICD-O-3 codes C50.0-C50.6, C50.8-C50.9) who received "systemic therapy before surgery" or "systemic therapy before and after surgery" (SEER item: 1639) were included. Subsequently, we identified patients with NAT records specific to the breast (SEER item: 1632), ensuring that all included patients underwent NAT for breast cancer. Fig. 1 illustrates the process of patient selection and the final number of patients included in the analysis. Ultimately, a cohort of 9968 breast cancer patients was included in this study. To minimize sample size loss, different population bases were used for analyzing two distinct long-term survival outcomes. Fifteen patients with unknown BCSS status were excluded, resulting in a final cohort of 9953 patients included for BCSS analysis. Our team accessed the SEER database, a public database that does not require informed consent. Approval for this study was obtained from the institutional review board of Zhuji Affiliated Hospital of Wenzhou Medical University (Approval No. 0103).

### Variables

We extracted and identified the following variables as covariates: (1) socio-demographic factors; (2) variables affecting reconstruction and surgical approaches or outcomes documented in previous studies; and (3) variables derived from our clinical practice. The complete adjustment model included the following variables: (1) continuous variables: age, number of lymph nodes examined, number of positive lymph nodes, months from diagnosis to treatment and year of diagnosis; (2) categorical variables: marital status, race, average household income, region of residence, response to NAT, quadrant of the primary site, TNM clinical stage groups according to the American Joint Committee on Cancer (AJCC) 8th edition, cT stage, cN stage, molecular subtype, estrogen receptor (ER) status, progesterone receptor (PR) status, human epidermal growth factor receptor 2 (HER-2) status, histology, laterality, post-mastectomy radiotherapy (PMRT), presence of carcinoma in situ (CIS), and pathological grade.

According to the SEER database definition, tumors with any amount of in situ carcinoma components were classified as including CIS. Breast cancer surgery after NAT included NSM, total (simple) mastectomy, modified radical mastectomy, radical mastectomy, and extended radical mastectomy. These procedures were categorized into two major groups based on the extent of mastectomy: NSM and TM. BR could be performed concurrently with mastectomy or at a later date. If delayed reconstruction was planned, a tissue expander would be inserted during the mastectomy procedure.

### Outcome measures

The primary outcomes of interest were OS and BCSS. OS was defined as the time interval from the date of diagnosis to the date of death from any cause, whereas BCSS was defined as the time interval from the date of diagnosis to the date of breast cancer-specific death.

### Statistical analysis

We compared continuous variables between groups using the independent samples *t*-test or Mann-Whitney *U test*, depending on data normality. Categorical data were analyzed using chi-square or Fisher's exact test as appropriate. Sensitivity analyses were conducted to evaluate the robustness of the findings. Missing covariate data were imputed using means or medians for continuous variables and NA or 9 for categorical variables. BR surgery performed after the interval following NAT introduces survival time bias, as only patients who survive this interval are eligible for surgery. To address this bias, we conducted a landmark analysis, excluding those who died or were lost to follow-up within 12 months of diagnosis. This analysis included 7794 patients. The association between each group and OS or BCSS was assessed using Cox proportional hazards models, with five models developed: a crude model; Model 1, adjusted for sociodemographic factors; Model 2, further adjusted for covariates that were significant (*P* < .10) or clinically relevant; Model 3, fully adjusted for all prespecified confounders; and Model 4, the landmark analysis. P value trends across surgical groups were evaluated to ensure result robustness. Kaplan-Meier (K-M) curves compared OS and BCSS among surgical groups, with log-rank tests for assessment. Interaction analysis for OS included 9968 patients and for BCSS included 9953 patients, stratified by subgroup variables, with likelihood ratio tests assessing subgroup interactions.

To balance baseline characteristics across treatment groups and to assess the impact of breast reconstruction on OS and BCSS, we paired participants in a 1:1 ratio via nearest-neighbor matching—repeated twice—with a caliper width set at 0.2 standard deviations of the logit-transformed propensity score to enhance baseline comparability. Individuals failing to satisfy prespecified inclusion criteria were omitted from the analysis. As part of sensitivity testing, we implemented four distinct propensity-based methods: propensity score adjustment (PSA), propensity score matching (PSM), inverse probability of treatment weighting (IPTW), and standardized mortality ratio weighting (SMRW). Effect estimates and p-values were recorded and compared across methods to evaluate consistency and robustness. All analyses used R (Version 4.2.2) and Free Statistics (Version 1.9.1). Results were considered statistically significant at *P* < .05.

## Results

### Baseline characteristics of selected participants

The baseline characteristics of the patients in this study are summarized in Table 1. A total of 9968 breast cancer patients meeting the selection criteria were included in the OS analysis, while 9953 patients were included in the BCSS analysis. As shown in Table 1, 4503 (45.2 %) patients opted for post-mastectomy BR, with 3554 (78.9 %) choosing TM with BR and 949 (21.1 %) selecting NSM with BR. The mean age of the total population was 50.7 ± 12.6 years. Patients in the NSM with BR group were younger, with a mean age of 45.8 ± 10.7 years, and had a higher percentage of individuals with household income >$75,000 (56.5 %). This group also had the highest rate of complete response (CR) to NAT at 47.3 %. Additionally, they had a higher proportion of patients with earlier clinical stages: stage Ⅰ (18.4 %) and stage Ⅱ (66.5 %), compared to the TM without BR group, which had stage Ⅰ (7.6 %) and stage Ⅱ (43.9 %). Regarding molecular subtypes, the NSM with BR group had a larger proportion of HR+/HER2+ (29.7 %) and HR-/HER2- (31.2 %) subtypes, whereas the TM without BR group had 21.7 % and 26.2 %, respectively. The NSM with BR group had the fewest lymph nodes removed postoperatively, with a median of 3.0 (IQR: 2.0–6.0), and 64.9 % of patients had pathological grade Ⅲ. Furthermore, this group had the lowest proportion of patients receiving post-mastectomy radiation therapy (PMRT) at 30.6 %, while the TM without BR group had the highest proportion at 55.8 %.

**Table 1:** Baseline characteristics of all included breast cancer patients.

Variables	Total (*n* = 9968)	TM without BR (*n* = 5465)	TM + BR (*n* = 3554)	NSM + BR (*n* = 949)	P-value
**Age, Mean ± SD**	50.7 ± 12.6	54.2 ± 13.0	46.5 ± 10.7	45.8 ± 10.7	< 0.001
**Marital status, n (%)**					0.007
Unmarried	2167 (21.7)	1222 (22.4)	749 (21.1)	196 (20.7)	
Married	7472 (75.0)	4035 (73.8)	2706 (76.1)	731 (77)	
Unknown	329 (3.3)	208 (3.8)	99 (2.8)	22 (2.3)	
**Race, n (%)**					< 0.001
Hispanic	2000 (20.1)	1168 (21.4)	659 (18.5)	173 (18.2)	
Non-Hispanic White	5261 (52.8)	2619 (47.9)	2073 (58.3)	569 (60)	
Non-Hispanic Black	1408 (14.1)	832 (15.2)	481 (13.5)	95 (10)	
Non-Hispanic Asian or Pacific Islander	1183 (11.9)	781 (14.3)	300 (8.4)	102 (10.7)	
Unknown	116 (1.2)	65 (1.2)	41 (1.2)	10 (1.1)	
**Average household income, n (%)**					< 0.001
<$50,000	887 (8.9)	610 (11.2)	237 (6.7)	40 (4.2)	
$50,000 - $75,000	4339 (43.5)	2511 (45.9)	1455 (40.9)	373 (39.3)	
>$75,000	4742 (47.6)	2344 (42.9)	1862 (52.4)	536 (56.5)	
**Region of residence**					< 0.001
Urban	9077 (91.1)	4872 (89.1)	3335 (93.8)	870 (91.7)	
Rural	869 (8.7)	577 (10.6)	213 (6)	79 (8.3)	
Unknown	22 (0.2)	16 (0.3)	6 (0.2)	0 (0)	
**Response to NAT, n (%)**					< 0.001
CR	3664 (36.8)	1757 (32.2)	1458 (41)	449 (47.3)	
PR	2966 (29.8)	1764 (32.3)	949 (26.7)	253 (26.7)	
NR	699 (7.0)	474 (8.7)	181 (5.1)	44 (4.6)	
CR or PR	2639 (26.5)	1470 (26.9)	966 (27.2)	203 (21.4)	
**Quadrant of primary site, n (%)**					< 0.001
Central quadrant	472 (4.7)	310 (5.7)	137 (3.9)	25 (2.6)	
Inner quadrant	1466 (14.7)	753 (13.8)	533 (15)	180 (19)	
Outer quadrant	4034 (40.5)	2135 (39.1)	1498 (42.1)	401 (42.3)	
Axillary tail	47 (0.5)	26 (0.5)	13 (0.4)	8 (0.8)	
Overlapping lesion	2306 (23.1)	1276 (23.3)	823 (23.2)	207 (21.8)	
Unknown quadrant	1643 (16.5)	965 (17.7)	550 (15.5)	128 (13.5)	
**TNM clinical stage groups AJCC (8th), n (%)**					< 0.001
ⅠA	1057 (10.6)	418 (7.6)	464 (13.1)	175 (18.4)	
ⅡA	2952 (29.6)	1288 (23.6)	1225 (34.5)	439 (46.3)	
ⅡB	2105 (21.1)	1108 (20.3)	805 (22.7)	192 (20.2)	
ⅢA	1710 (17.2)	1020 (18.7)	608 (17.1)	82 (8.6)	
ⅢB	1130 (11.3)	926 (16.9)	184 (5.2)	20 (2.1)	
ⅢC	1014 (10.2)	705 (12.9)	268 (7.5)	41 (4.3)	
**cT stage, n (%)**					< 0.001
cT0	17 (0.2)	10 (0.2)	4 (0.1)	3 (0.3)	
cT1	1697 (17.0)	764 (14)	711 (20)	222 (23.4)	
cT2	4688 (47.0)	2248 (41.1)	1863 (52.4)	577 (60.8)	
cT3	2085 (20.9)	1228 (22.5)	734 (20.7)	123 (13)	
cT4	1481 (14.9)	1215 (22.2)	242 (6.8)	24 (2.5)	
**cN stage, n (%)**					< 0.001
cN0	4422 (44.4)	2012 (36.8)	1769 (49.8)	641 (67.5)	
cN1	3414 (34.2)	2006 (36.7)	1194 (33.6)	214 (22.6)	
cN2	1118 (11.2)	742 (13.6)	323 (9.1)	53 (5.6)	
cN3	1014 (10.2)	705 (12.9)	268 (7.5)	41 (4.3)	
**Molecular subtype, n (%)**					< 0.001
HR+/HER-2+	2465 (24.7)	1188 (21.7)	995 (28)	282 (29.7)	
HR+/HER-2-	3210 (32.2)	1907 (34.9)	1044 (29.4)	259 (27.3)	
HR-/HER-2+	1357 (13.6)	805 (14.7)	454 (12.8)	98 (10.3)	
HR-/HER-2-	2736 (27.4)	1432 (26.2)	1008 (28.4)	296 (31.2)	
Unknown	200 (2.0)	133 (2.4)	53 (1.5)	14 (1.5)	
**ER status, n (%)**					0.561
Positive	5492 (55.1)	3012 (55.1)	1964 (55.3)	516 (54.4)	
Negative	4415 (44.3)	2414 (44.2)	1571 (44.2)	430 (45.3)	
Unknown	61 (0.6)	39 (0.7)	19 (0.5)	3 (0.3)	
**PR status, n (%)**					0.139
Positive	4181 (41.9)	2271 (41.6)	1523 (42.9)	387 (40.8)	
Negative	5715 (57.3)	3145 (57.5)	2013 (56.6)	557 (58.7)	
Unknown	72 (0.7)	49 (0.9)	18 (0.5)	5 (0.5)	
**HER-2 status, n (%)**					< 0.001
Positive	3831 (38.4)	1997 (36.5)	1452 (40.9)	382 (40.3)	
Negative	5959 (59.8)	3347 (61.2)	2055 (57.8)	557 (58.7)	
Unknown	178 (1.8)	121 (2.2)	47 (1.3)	10 (1.1)	
**Histology, n (%)**					< 0.001
IBC—NST	9110 (91.4)	4897 (89.6)	3313 (93.2)	900 (94.8)	
IBC-ST	760 (7.6)	502 (9.2)	218 (6.1)	40 (4.2)	
Rare and SGT	98 (1.0)	66 (1.2)	23 (0.6)	9 (0.9)	
**Laterality, n (%)**					0.627
Left side	5066 (50.8)	2800 (51.2)	1793 (50.5)	473 (49.8)	
Right side	4902 (49.2)	2665 (48.8)	1761 (49.5)	476 (50.2)	
**Number of lymph nodes examined, Median (IQR)**	6.0 (3.0, 12.0)	7.0 (3.0, 14.0)	4.5 (2.0, 10.0)	3.0 (2.0, 6.0)	< 0.001
**Number of positive lymph nodes, Median (IQR)**	0.0 (0.0, 1.0)	0.0 (0.0, 1.0)	0.0 (0.0, 1.0)	0.0 (0.0, 0.0)	< 0.001
**Months from diagnosis to treatment, Median (IQR)**	1.0 (1.0, 1.0)	1.0 (1.0, 1.0)	1.0 (0.0, 1.0)	1.0 (1.0, 1.0)	< 0.001
**Year of diagnosis, Median (IQR)**	2018 (2015, 2019)	2018 (2014, 2019)	2018 (2015, 2019)	2018 (2016, 2019)	< 0.001
**PMRT, n (%)**					< 0.001
Yes	4898 (49.1)	3049 (55.8)	1559 (43.9)	290 (30.6)	
No	5070 (50.9)	2416 (44.2)	1995 (56.1)	659 (69.4)	
**Presence of CIS, n (%)**					< 0.001
No	2005 (20.1)	1233 (22.6)	642 (18.1)	130 (13.7)	
Yes	1640 (16.5)	882 (16.1)	654 (18.4)	104 (11)	
Unknown	6323 (63.4)	3350 (61.3)	2258 (63.5)	715 (75.3)	
**Pathological grade, n (%)**					< 0.001
Ⅰ	386 (3.9)	231 (4.2)	124 (3.5)	31 (3.3)	
Ⅱ	2973 (29.8)	1686 (30.9)	1019 (28.7)	268 (28.2)	
Ⅲ	5945 (59.6)	3113 (57)	2216 (62.4)	616 (64.9)	
Unknown	664 (6.7)	435 (8)	195 (5.5)	34 (3.6)	

BR breast reconstruction; TM total mastectomy; NSM nipple-sparing mastectomy; NAT neoadjuvant therapy; CR complete response; PR partial response; NR no response; ER estrogen receptor; PR progesterone receptor; HER-2 human epidermal growth factor receptor 2; HR hormone receptor; IBC—NST invasive breast carcinoma of no special type; IBC-ST invasive breast carcinoma of special type; SGT salivary gland-type; PMRT postmastectomy radiotherapy; CIS carcinoma in situ.

### Univariate and multi-model multivariate analysis

Table 2 presents the univariate analysis of various covariates with OS and BCSS in breast cancer patients receiving NAT. The results showed that compared with TM patients who did not undergo BR, those who received BR were associated with better OS (HR: 0.56; 95 % CI: 0.48–0.64; *P* < .001) and BCSS (HR: 0.65; 95 % CI: 0.56–0.75; *P* < .001). Similarly, among patients who underwent NSM, BR was associated with improved OS (HR: 0.29; 95 % CI: 0.20–0.42; *P* < .001) and BCSS (HR: 0.31; 95 % CI: 0.20–0.46; *P* < .001). Non-Hispanic Asian or Pacific Islander ethnicity, average household income >$75,000, and PMRT were protective factors for both OS and BCSS (*P* < .05). Conversely, age ≥65 years, partial or no response (PR/NR) to NAT, TNM clinical stages Ⅱ or Ⅲ, ER-, PR-, HER2-, special types of invasive breast carcinoma (IBC-ST), salivary gland-type histology (SGT), >10 lymph nodes examined, four or more positive lymph nodes, and pathological grade Ⅲ were associated with poorer OS and BCSS (*P* < .05).

**Table 2:** Univariate COX analysis of each variable with respect to OS and BCSS.

Variable	OS				BCSS			
	Total (*n* = 9968)	Event (%)	Crude HR (95 %CI)	P-value	Total (*n* = 9953)	Event (%)	Crude HR (95 %CI)	P-value
**Surgical procedure**								
TM without BR	5465	703 (12.9)	1(Ref)		5454	562 (10.3)	1(Ref)	
TM + BR	3554	259 (7.3)	0.56 (0.48∼0.64)	<0.001	3550	240 (6.8)	0.65 (0.56∼0.75)	<0.001
NSM + BR	949	28 (3.0)	0.29 (0.20∼0.42)	<0.001	949	24 (2.5)	0.31 (0.20∼0.46)	<0.001
**Age**								
<65 yr	8456	774 (9.2)	1(Ref)		8445	687 (8.1)	1(Ref)	
≥65 yr	1512	216 (14.3)	1.78 (1.53∼2.07)	<0.001	1508	139 (9.2)	1.29 (1.07∼1.55)	0.007
**Marital status**								
Unmarried	2167	226 (10.4)	1(Ref)		2161	189 (8.7)	1(Ref)	
Married	7472	732 (9.8)	0.89 (0.77∼1.03)	0.126	7463	608 (8.1)	0.88 (0.75∼1.04)	0.130
Unknown	329	32 (9.7)	0.94 (0.65∼1.36)	0.738	329	29 (8.8)	1.01 (0.68∼1.50)	0.954
**Race**								
Hispanic	2000	186 (9.3)	1(Ref)		1995	162 (8.1)	1(Ref)	
Non-Hispanic White	5261	531 (10.1)	0.93 (0.79∼1.10)	0.389	5256	437 (8.3)	0.88 (0.73∼1.05)	0.157
Non-Hispanic Black	1408	176 (12.5)	1.22 (0.99∼1.50)	0.060	1405	143 (10.2)	1.14 (0.91∼1.43)	0.257
Non-Hispanic Asian or Pacific Islander	1183	87 (7.4)	0.74 (0.57∼0.95)	0.020	1181	76 (6.4)	0.74 (0.56∼0.97)	0.031
Unknown	116	10 (8.6)	0.78 (0.41∼1.48)	0.450	116	8 (6.9)	0.72 (0.35∼1.46)	0.362
**Average household income, n (%)**								
<$50,000	887	126 (14.2)	1(Ref)		884	100 (11.3)	1(Ref)	
$50,000 - $75,000	4339	508 (11.7)	0.79 (0.65∼0.96)	0.019	4331	422 (9.7)	0.83 (0.67∼1.03)	0.091
>$75,000	4742	356 (7.5)	0.71 (0.58∼0.87)	0.001	4738	304 (6.4)	0.76 (0.60∼0.95)	0.016
**Region of residence**								
Urban	9077	877 (9.7)	1(Ref)		9065	741 (8.2)	1(Ref)	
Rural	869	110 (12.7)	1.34 (1.10∼1.64)	0.003	866	83 (9.6)	1.20 (0.96∼1.51)	0.111
Unknown	22	3 (13.6)	1.64 (0.53∼5.10)	0.392	22	2 (9.1)	1.28 (0.32∼5.13)	0.727
**Response to NAT**								
CR	3664	158 (4.3)	1(Ref)		3662	131 (3.6)	1(Ref)	
PR	2966	427 (14.4)	3.40 (2.83∼4.08)	<0.001	2959	354 (12)	3.41 (2.79∼4.16)	<0.001
NR	699	115 (16.5)	6.57 (5.16∼8.36)	<0.001	698	100 (14.3)	6.87 (5.29∼8.92)	<0.001
CR or PR	2639	290 (11)	3.07 (2.52∼3.72)	<0.001	2634	241 (9.1)	3.07 (2.48∼3.80)	<0.001
**Quadrant of primary site**								
Central quadrant	472	46 (9.7)	1(Ref)		470	35 (7.4)	1(Ref)	
Inner quadrant	1466	109 (7.4)	0.90 (0.63∼1.26)	0.529	1463	87 (5.9)	0.94 (0.63∼1.38)	0.739
Outer quadrant	4034	363 (9)	1.02 (0.75∼1.39)	0.889	4032	307 (7.6)	1.13 (0.80∼1.60)	0.490
Axillary tail	47	6 (12.8)	1.29 (0.55∼3.02)	0.555	47	6 (12.8)	1.69 (0.71∼4.02)	0.235
Overlapping lesion	2306	243 (10.5)	1.21 (0.88∼1.66)	0.238	2303	200 (8.7)	1.30 (0.91∼1.86)	0.151
Unknown quadrant	1643	223 (13.6)	1.50 (1.09∼2.06)	0.013	1638	191 (11.7)	1.68 (1.17∼2.41)	0.005
**TNM clinical stage groups AJCC (8th)**								
ⅠA	1057	18 (1.7)	1(Ref)		1056	8 (0.8)	1(Ref)	
ⅡA	2952	122 (4.1)	1.98 (1.21∼3.25)	0.007	2948	94 (3.2)	3.43 (1.67∼7.07)	0.001
ⅡB	2105	166 (7.9)	2.74 (1.68∼4.45)	<0.001	2102	131 (6.2)	4.88 (2.39∼9.97)	<0.001
ⅢA	1710	225 (13.2)	4.74 (2.93∼7.66)	<0.001	1709	197 (11.5)	9.39 (4.63∼19.05)	<0.001
ⅢB	1130	232 (20.5)	7.17 (4.44∼11.59)	<0.001	1126	196 (17.4)	13.72 (6.76∼27.83)	<0.001
ⅢC	1014	227 (22.4)	10.57 (6.54∼17.09)	<0.001	1012	200 (19.8)	21.02 (10.37∼42.63)	<0.001
**cT stage**								
cT0	17	0 (0)	NA	NA	17	0 (0)	NA	NA
cT1	1697	61 (3.6)	1(Ref)		1695	43 (2.5)	1(Ref)	
cT2	4688	319 (6.8)	1.64 (1.25∼2.16)	<0.001	4681	266 (5.7)	1.94 (1.41∼2.68)	<0.001
cT3	2085	266 (12.8)	2.73 (2.07∼3.61)	<0.001	2085	227 (10.9)	3.31 (2.39∼4.59)	<0.001
cT4	1481	344 (23.2)	4.98 (3.79∼6.54)	<0.001	1475	290 (19.7)	5.99 (4.34∼8.25)	<0.001
**cN stage**								
cN0	4422	176 (4)	1(Ref)		4418	126 (2.9)	1(Ref)	
cN1	3414	421 (12.3)	1.88 (1.57∼2.24)	<0.001	3406	349 (10.2)	2.19 (1.78∼2.69)	<0.001
cN2	1118	166 (14.8)	3.28 (2.65∼4.06)	<0.001	1117	151 (13.5)	4.19 (3.31∼5.31)	<0.001
cN3	1014	227 (22.4)	5.17 (4.24∼6.29)	<0.001	1012	200 (19.8)	6.38 (5.10∼7.98)	<0.001
**Molecular subtype**								
HR+/HER-2+	2465	132 (5.4)	1(Ref)		2464	109 (4.4)	1(Ref)	
HR+/HER-2-	3210	368 (11.5)	2.26 (1.85∼2.76)	<0.001	3205	300 (9.4)	2.24 (1.80∼2.79)	<0.001
HR-/HER-2+	1357	101 (7.4)	1.30 (1.00∼1.68)	0.050	1349	75 (5.6)	1.17 (0.87∼1.57)	0.294
HR-/HER-2-	2736	364 (13.3)	2.91 (2.38∼3.55)	<0.001	2735	321 (11.7)	3.11 (2.50∼3.86)	<0.001
Unknown	200	25 (12.5)	1.89 (1.23∼2.90)	0.004	200	21 (10.5)	1.93 (1.21∼3.08)	0.006
**ER status**								
Positive	5492	474 (8.6)	1(Ref)		5487	387 (7.1)	1(Ref)	
Negative	4415	505 (11.4)	1.39 (1.22∼1.57)	<0.001	4405	431 (9.8)	1.45 (1.26∼1.66)	<0.001
Unknown	61	11 (18.0)	2.21 (1.22∼4.02)	0.009	61	8 (13.1)	1.97 (0.98∼3.97)	0.057
**PR status**								
Positive	4181	321 (7.7)	1(Ref)		4177	258 (6.2)	1(Ref)	
Negative	5715	657 (11.5)	1.57 (1.38∼1.80)	<0.001	5704	560 (9.8)	1.67 (1.44∼1.93)	<0.001
Unknown	72	12 (16.7)	1.90 (1.07∼3.38)	0.029	72	8 (11.1)	1.58 (0.78∼3.19)	0.204
**HER-2 status**								
Positive	3831	234 (6.1)	1(Ref)		3822	184 (4.8)	1(Ref)	
Negative	5959	734 (12.3)	2.29 (1.98∼2.65)	<0.001	5953	622 (10.4)	2.47 (2.09∼2.91)	<0.001
Unknown	178	22 (12.4)	1.65 (1.06∼2.55)	0.025	178	20 (11.2)	1.91 (1.20∼3.03)	0.006
**Histology**								
IBC—NST	9110	862 (9.5)	1(Ref)		9095	718 (7.9)	1(Ref)	
IBC-ST	760	105 (13.8)	1.49 (1.22∼1.83)	<0.001	760	87 (11.4)	1.48 (1.19∼1.85)	0.001
Rare and SGT	98	23 (23.5)	3.53 (2.34∼5.35)	<0.001	98	21 (21.4)	3.86 (2.50∼5.95)	<0.001
**Laterality**								
Left side	5066	501 (9.9)	1(Ref)		5057	418 (8.3)	1(Ref)	
Right side	4902	489 (10)	1.00 (0.89∼1.14)	0.943	4896	408 (8.3)	1.00 (0.88∼1.15)	0.954
**Number of lymph nodes examined**								
≤10	6848	532 (7.8)	1(Ref)		6840	442 (6.5)	1(Ref)	
>10	3120	458 (14.7)	1.57 (1.38∼1.77)	<0.001	3113	384 (12.3)	1.59 (1.38∼1.82)	<0.001
**Number of positive lymph nodes**								
0–3	8539	687 (8)	1(Ref)		8526	559 (6.6)	1(Ref)	
4–9	1032	198 (19.2)	3.53 (3.01∼4.14)	<0.001	1031	173 (16.8)	3.80 (3.20∼4.50)	<0.001
≥10	397	105 (26.4)	6.63 (5.39∼8.16)	<0.001	396	94 (23.7)	7.29 (5.85∼9.09)	<0.001
**Months from diagnosis to treatment**								
≤1 mon	7706	797 (10.3)	1(Ref)		7694	676 (8.8)	1(Ref)	
>1 mon	2262	193 (8.5)	0.91 (0.78∼1.07)	0.256	2259	150 (6.6)	0.84 (0.70∼1.00)	0.047
**Year of diagnosis**								
2010–2015	2970	610 (20.5)	1(Ref)		2960	513 (17.3)	1(Ref)	
2016–2020	6998	380 (5.4)	0.89 (0.77∼1.03)	0.118	6993	313 (4.5)	0.85 (0.73∼0.99)	0.040
**PMRT**								
Yes	4898	635 (13)	1(Ref)		4889	552 (11.3)	1(Ref)	
No	5070	355 (7)	0.66 (0.58∼0.75)	<0.001	5064	274 (5.4)	0.58 (0.50∼0.67)	<0.001
**Presence of CIS**								
No	2005	334 (16.7)	1(Ref)		2000	271 (13.6)	1(Ref)	
Yes	1640	280 (17.1)	1.02 (0.87∼1.20)	0.81	1636	242 (14.8)	1.09 (0.91∼1.29)	0.346
Unknown	6323	376 (5.9)	1.01 (0.87∼1.18)	0.86	6317	313 (5)	1.04 (0.88∼1.23)	0.668
**Pathological grade**								
Ⅰ	386	22 (5.7)	1(Ref)		386	14 (3.6)	1(Ref)	
Ⅱ	2973	241 (8.1)	1.43 (0.93∼2.22)	0.107	2968	183 (6.2)	1.71 (0.99∼2.95)	0.052
Ⅲ	5945	646 (10.9)	1.89 (1.24∼2.90)	0.003	5936	558 (9.4)	2.57 (1.51∼4.37)	<0.001
Unknown	664	81 (12.2)	1.46 (0.91∼2.34)	0.117	663	71 (10.7)	2.02 (1.14∼3.58)	0.016

OS overall survival; BCSS breast cancer-specific survival; BR breast reconstruction; TM total mastectomy; NSM nipple-sparing mastectomy; NAT neoadjuvant therapy; CR complete response; PR partial response; NR no response; ER estrogen receptor; PR progesterone receptor; HER-2 human epidermal growth factor receptor 2; HR hormone receptor; IBC—NST invasive breast carcinoma of no special type; IBC-ST invasive breast carcinoma of special type; SGT salivary gland-type; PMRT postmastectomy radiotherapy; CIS carcinoma in situ; HR Hazard ratios; CI Confidence intervals.

We conducted multivariate Cox proportional hazards models to evaluate the impact of BR and various surgical approaches on OS and BCSS in patients who underwent NAT for breast cancer (Table 3). Compared with TM patients who did not undergo BR, those who received BR were associated with better OS across all three multivariable models (Model 1–3, HR: 0.64–0.85; all *P* < .05; Table 3). In contrast, the landmark analysis showed no statistically significant association (HR: 0.89, 95 % CI: 0.75–1.05; *P* = .161). However, among patients who underwent NSM, BR was associated with improved OS across all models compared with those who underwent TM alone (HR: 0.29–0.60; all *P* < .05; Table 3). Similarly, in Models 2–4, TM patients who received BR showed no significant improvement in BCSS compared with those who did not undergo BR (HR: 0.90–0.96; all *P* > .05; Table 3). However, in all models, patients who received NSM combined with BR treatment were associated with better BCSS compared with those who received TM alone (HR: 0.31–0.64; all *P* < .05; Table 3). Sensitivity analysis revealed a statistically significant trend across models (*P* < .05) except for Model 4, suggesting potential instability in the findings and warranting further validation (Table 3).

**Table 3:** Multivariate COX analysis of the association between different surgical modalities and OS, BCSS.

OS										
Variable	Crude mode		Multivariable-adjusted model 1		Multivariable-adjusted model 2		Multivariable-adjusted model 3		Landmark analysis model 4	
	HR (95 %CI)	*P* -value	HR (95 %CI)	*P* -value	HR (95 %CI)	*P* -value	HR (95 %CI)	*P* -value	HR (95 %CI)	*P* -value
Trend test^1^	0.55 (0.49∼0.62)	< 0.001	0.61 (0.54∼0.70)	< 0.001	0.81 (0.71∼0.92)	0.001	0.80 (0.70∼0.91)	0.001	0.84 (0.73∼0.97)	0.015
TM without BR	1 (Ref)		1 (Ref)		1 (Ref)		1 (Ref)		1 (Ref)	
TM + BR	0.56 (0.48∼0.64)	< 0.001	0.64 (0.55∼0.74)	<0.001	0.85 (0.72∼0.99)	0.036	0.83 (0.71∼0.97)	0.019	0.89 (0.75∼1.05)	0.161
NSM + BR	0.29 (0.20∼0.42)	< 0.001	0.34 (0.23∼0.49)	<0.001	0.57 (0.39∼0.85)	0.005	0.57 (0.39∼0.85)	0.005	0.60 (0.39∼0.91)	0.017
**BCSS**										
Trend test^1^	0.61 (0.54∼0.69)	< 0.001	0.63 (0.55∼0.72)	<0.001	0.86 (0.75∼0.99)	0.033	0.84 (0.73∼0.97)	0.017	0.90 (0.77∼1.04)	0.139
TM without BR	1 (Ref)		1 (Ref)		1 (Ref)		1 (Ref)		1 (Ref)	
TM + BR	0.65 (0.56∼0.75)	< 0.001	0.67 (0.57∼0.79)	<0.001	0.92 (0.78∼1.09)	0.352	0.90 (0.76∼1.06)	0.202	0.96 (0.81∼1.15)	0.660
NSM + BR	0.31 (0.20∼0.46)	< 0.001	0.32 (0.21∼0.49)	<0.001	0.59 (0.39∼0.90)	0.015	0.59 (0.39∼0.90)	0.014	0.64 (0.41∼0.98)	0.048

1A test for linear trend was performed for each model.

Multivariable-adjusted model 1: Adjusted for age, marital status, race, average household income and region of residence;.

Multivariable-adjusted model 2: Adjusted for age, marital status, race, average household income, region of residence, response to NAT, TNM clinical stage groups AJCC (8th), ER status, PR status, HER-2 status, histology, number of drained lymph nodes, number of positive lymph nodes, PMRT, pathological grade, and year of diagnosis;.

Multivariable-adjusted model 3: Adjusted for age, marital status, race, average household income, region of residence, response to NAT, TNM clinical stage groups AJCC (8th), ER status, PR status, HER-2 status, histology, number of drained lymph nodes, number of positive lymph nodes, PMRT, pathological grade, year of diagnosis, quadrant of primary site, laterality, months from diagnosis to treatment and composition of CIS.

OS overall survival; BCSS breast cancer-specific survival; BR breast reconstruction; TM total mastectomy; NSM nipple-sparing mastectomy; NAT neoadjuvant therapy; ER estrogen receptor; PR progesterone receptor; HER-2 human epidermal growth factor receptor 2; PMRT postmastectomy radiotherapy; CIS carcinoma in situ; HR Hazard ratios; CI Confidence intervals.

### Propensity score matching and multiple association inference models

Baseline characteristic analysis revealed that patients who underwent NSM combined with BR were younger, had earlier disease stages, higher rates of negative lymph node status, and better responses to NAT than those in the comparison group. To further evaluate the impact of different surgical approaches on OS and BCSS, PSM and multivariable regression models were used. The matched cohorts comprised 5412 and 1758 patients, respectively; the standardized mean difference (SMD) for all variables was <0.1 in both groups, indicating successful balance of covariates after matching (Supplementary Table S1 and S2). The results indicated that, following PSM, none of the four association inference models (all SMD < 0.1; Supplementary Figures S1) revealed a statistically significant survival advantage—either in OS or BCSS—for patients receiving TM combined with BR, compared with those receiving TM alone (OR: 0.84–1.00; all *P* > .05; Table 4). Similarly, when comparing patients who underwent NSM combined with BR to those who received TM alone, IPTW analysis showed no statistically significant association with OS (OR: 1.10; *P* = .369; Table 4) or BCSS (OR: 1.12; *P* = .385; Table 4). However, in the remaining three analytical models, patients who underwent NSM combined with BR showed statistically significant improvements in OS (OR: 0.58–0.62; all *P* < .05; Table 4) and BCSS (OR: 0.56–0.63; all *P* < .05; Table 4). We observed that different bias-correction methods yielded apparently discrepant results. A thorough investigation revealed that the estimated treatment effect was not absent, but rather obscured by inadequate performance of IPTW (SMD > 0.1; Supplementary Figures S2) under substantial covariate imbalance. In contrast, other more robust methods—including PSM and SMRW—yielded consistent and statistically significant associations with improved survival outcomes.

**Table 4:** OS and BCSS for four multiple association inference models are presented across treatment groups after PSM.

Models	Item	OS		BCSS	
		OR (95 %CI)	P-value	OR (95 %CI)	P-value
Propensity score adjusted	TM + BR vs. TM without BR	0.86 (0.73∼1.02)	0.075	0.94 (0.79∼1.12)	0.508
Propensity score matching	TM + BR vs. TM without BR	0.94 (0.78∼1.14)	0.559	1.00 (0.82∼1.22)	1.000
Propensity score IPTW	TM + BR vs. TM without BR	0.84 (0.73∼0.97)	0.018	0.93 (0.80∼1.08)	0.352
Propensity score SMRW	TM + BR vs. TM without BR	0.89 (0.76∼1.05)	0.157	0.96 (0.81∼1.14)	0.648
Propensity score adjusted	NSM + BR vs. TM without BR	0.60 (0.40∼0.90)	0.014	0.62 (0.40∼0.95)	0.030
Propensity score matching	NSM + BR vs. TM without BR	0.58 (0.36∼0.94)	0.027	0.56 (0.34∼0.93)	0.026
Propensity score IPTW	NSM + BR vs. TM without BR	1.10 (0.90∼1.35)	0.369	1.12 (0.98∼1.36)	0.385
Propensity score SMRW	NSM + BR vs. TM without BR	0.62 (0.42∼0.92)	0.018	0.63 (0.41∼0.96)	0.032

(1)propensity score adjusted (PSA); (2) propensity score matching (PSM); (3) inverse probability of treatment weighting (IPTW) regression analysis; (4) standardised mortality ratio weighting (SMRW) regression analysis.

OS overall survival; BCSS breast cancer-specific survival; BR breast reconstruction; TM total mastectomy; NSM nipple-sparing mastectomy; OR Odds ratios; CI Confidence intervals.

### K-M survival curves and outcomes

**Fig. 2:**
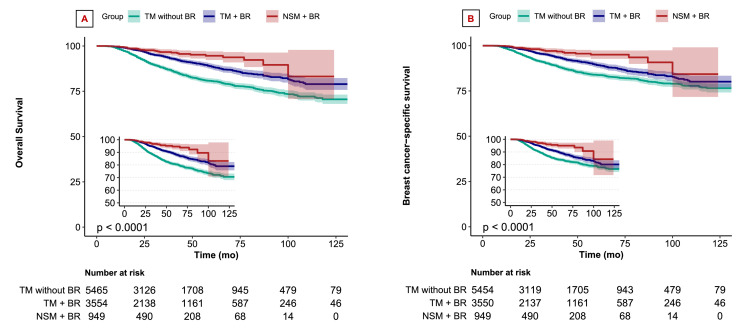
Kaplan-Meier curves of OS (A) and BCSS (B) for breast cancer patients across three surgical approach groups.

To further investigate the impact of the three different surgical approaches on OS and BCSS, we constructed K-M survival curves (Fig. 2). The median follow-up time for the entire cohort was 31.9 months. The 5-year OS rates were as follows: 80.6 % for the TM without BR group, 88.6 % for the TM with BR group, and 94.6 % for the NSM with BR group (Fig. 2). The 5-year BCSS rates were 83.8 % for the TM without BR group, 89.3 % for the TM with BR group, and 95.0 % for the NSM with BR group (Fig. 2). The 10-year OS and BCSS rates were 70.6 % and 76.5 % for the TM without BR group, 79.0 % and 80.1 % for the TM with BR group, and 83.2 % and 84.3 % for the NSM with BR group (*P* < .001; Fig. 2).

### Subgroup analysis

**Fig. 3:**
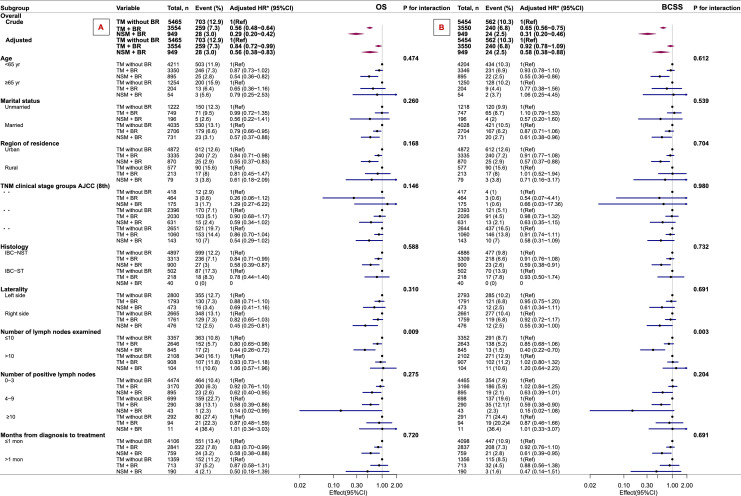
Subgroup analysis of OS (A) and BCSS (B) in breast cancer patients across three surgical approach groups. *We conducted subset analyses adjusted for Model 2 for the two long-term outcomes of breast cancer (OS and BCSS). OS overall survival; BCSS breast cancer-specific survival; NAT neoadjuvant therapy; ER estrogen receptor; PR progesterone receptor; HER-2 human epidermal growth factor receptor 2; PMRT postmastectomy radiotherapy; HR Hazard ratios; CI Confidence intervals.

In the entire cohort, we conducted subset analyses adjusted for Model 2 to evaluate the long-term outcomes (OS and BCSS) of breast cancer (Fig. 3). We examined the trend of effect sizes for all covariates as stratification factors and identified the covariates significantly associated with the outcomes: age, marital status, region of residence, TNM clinical stage group, histology, laterality, number of lymph nodes examined, number of positive lymph nodes, and months from diagnosis to treatment. According to our pre-specified criteria (P values for all interactions < 0.05), significant interactions were observed only in the number of lymph nodes examined group (*P* < .05). This suggests that in patients with >10 lymph nodes examined, surgical approaches of TM with BR or NSM with BR do not improve long-term outcomes but are not inferior to TM without BR (OS: TM with BR, HR: 0.93; 95 % CI: 0.73–1.18; NSM with BR, HR: 1.06; 95 % CI: 0.57–1.96; BCSS: TM with BR, HR: 1.02; 95 % CI: 0.80–1.32; NSM with BR, HR: 1.20; 95 % CI: 0.64–2.23; Fig. 3). Correspondingly, in the subgroups where NSM combined with BR was associated with improved OS and BCSS compared with TM without BR, the patient- and tumor-related characteristics were as follows: age < 65 years, married status, IBC—NST, right-sided tumor location, ≤10 lymph nodes examined, 0–3 positive lymph nodes, and a diagnosis-to-treatment interval ≤1 month (HR values are shown in Fig. 3). For patients with different clinical stages, the surgical approach of NSM with BR showed a trend toward survival benefit (stage Ⅱ: OS, HR: 0.59; 95 % CI: 0.34–1.02; BCSS, HR: 0.63; 95 % CI: 0.35–1.15; stage Ⅲ: OS, HR: 0.54; 95 % CI: 0.29–1.02; BCSS: HR: 0.58; 95 % CI: 0.31–1.09).

## Discussion

Our study is the first large-sample report in the United States to examine the long-term survival of female breast cancer patients undergoing BR and different surgical approaches after NAT. Our findings demonstrate that patients who underwent TM combined with BR showed no statistically significant difference in outcomes compared with those who underwent TM alone; in contrast, patients who underwent NSM combined with BR were associated with better OS and BCSS. Furthermore, through subgroup analysis, we identified specific patient subgroups more likely to benefit from NSM combined with BR. These results suggest that personalized surgical approaches should be adopted, reflecting the heterogeneity of the disease and the diverse needs of patients.

The safety and efficacy of reconstruction techniques following TM and NSM have been well-established. In the early context of non-neoadjuvant therapy, these studies indeed observed a survival benefit in patients undergoing breast reconstruction compared to those undergoing simple mastectomy alone [[21], [22], [23]]. Platt J et al. found that breast reconstruction was associated with a 17 % reduction in the risk of death and a 19 % reduction in the risk of breast cancer-specific mortality (*P* < .05). However, this study did not collect clinical data regarding breast cancer staging and molecular typing, and the results should be interpreted with caution [24]. Similarly, Bezuhly M et al. reported that immediate breast reconstruction was associated with a reduction in breast cancer-specific mortality, particularly in younger women. This association was likely influenced by socio-economic factors and disparities in care access [25]. Our findings align with these results but extend them by considering NSM in the context of neoadjuvant therapy (NAT), socio-economic status, and residential environment, thereby addressing key limitations in previous studies and enhancing the reliability of our results.

With the advancement of NAT in breast cancer treatment, the risks and outcomes of reconstruction following mastectomy warrant re-evaluation. Regarding surgical risks and complications, Gang Li et al. found in a meta-analysis that immediate breast reconstruction (IBR) after TM did not affect postoperative survival rates in patients receiving NAT, although it slightly increased short-term surgical complications without significant long-term differences [26]. Warren Peled A et al. reported lower complication rates for the nipple-areola complex (NAC), reduced nipple involvement, and lower local recurrence rates with NSM [27]. Burdge EC et al. concluded that both skin-sparing mastectomy (SSM) and NSM can be offered to advanced cancer patients requiring postoperative radiotherapy, with comparable complication rates to traditional mastectomy [28]. From an oncological perspective, Wu ZY et al. found similar local recurrence-free survival (LRFS), disease-free survival (DFS), distant metastasis-free survival (DMFS), and OS rates between IBR with NSM/SSM and simple mastectomy after NAT, supporting the feasibility of IBR with NSM/SSM [[15], [16]]. Ryu JM et al. observed no significant differences in OS, DFS, DMFS, and LRFS between IBR after SSM or NSM and simple mastectomy in a small Korean sample with short follow-up [17]. Prabhu R et al. noted a tendency towards survival benefits, likely due to the short follow-up and limited sample size [18]. Vieira RADC et al. found higher progression-free survival (PFS) in the IBR group after NSM compared to the TM group in locally advanced breast cancer (LABC) patients (average 88.8 months vs. 73.7 months; *P* = .05) [19]. Overall, multiple studies support our findings: adjusting for other factors, NSM with BR reduces the total risk of death by 43 % and the risk of breast cancer-specific death by 41 % (Model 3; OS, HR: 0.57; *P* = .005; BCSS, HR: 0.59; *P* = .014; Table 3).

The impact of NAT on surgical complications and survival outcomes is a critical consideration in treatment planning. Zhang C et al. found that NAT does not increase the risk of surgical site infection after IBR [9]. Several studies show that short-term complications associated with NAT and NSM reconstruction are comparable to those following adjuvant chemotherapy, making this combined approach viable for specific patient groups [[10], [11], [12], [13]]. Liu CH et al. reported similar long-term OS between patients undergoing IBR after neoadjuvant chemotherapy and those receiving adjuvant chemotherapy plus targeted therapy for early breast cancer [29]. These findings support the feasibility of mastectomy with reconstruction following NAT, including NSM. Our study also confirms the viability of reconstruction after NAT. Recent attention has focused on breast reconstruction following neoadjuvant chemoradiotherapy, which offers advantages such as shorter treatment time and better cosmetic outcomes [[30], [31]]. This sequence can increase the rate of IBR without reducing pathologic complete response (pCR) rates and should be considered an acceptable treatment option [32]. It does not appear to increase IBR complications or affect DFS and OS [33]. Furthermore, in locally advanced breast cancer, mastectomy followed by immediate autologous breast reconstruction (IABR) after neoadjuvant chemoradiotherapy is safe and may not compromise oncological outcomes [34]. Future studies should further explore these findings.

Due to the diverse biological behaviors of breast cancer, socio-economic factors influencing patient choices, and advancements in oncological and plastic surgery techniques, personalized surgery is crucial. Our study indicates that specific subgroups benefit more from certain surgical methods, aligning with tailored treatment strategies advocated in the literature. Wu ZY et al. found that among young female breast cancer patients receiving NAT, those undergoing NSM with BR had significantly lower breast cancer mortality (14.9% vs. 27.2 %; *P* = .023) and improved BCSS (89.1 % vs. 77.6 %; *P* = .048) compared to those undergoing simple TM [35]. Aurilio G et al. reported that in ER- patients post-NAT, IBR following TM was associated with higher local recurrence rates but did not affect OS, PFS, or distant metastasis rates [36]. Park S et al. found no differences in OS between patients undergoing IBR with TM and TM alone, regardless of pathologic complete response (pCR) status [37], consistent with our findings. Our study further explored subgroup heterogeneity and identified that, among patients who underwent NSM combined with BR, age, marital status, pathological type, tumor location, number of lymph nodes examined, and number of positive lymph nodes were significantly associated with improved survival outcomes. The survival benefits in these subgroups are likely due to the combined effects of effective tumor treatment and socio-psychological impacts. Compared to simple mastectomy, successful BR can enhance patient satisfaction, socio-psychological health, and quality of life, particularly for early-stage patients with a higher likelihood of long-term survival [[38], [39]].

In our study, although we adjusted for key clinical and socioeconomic factors, residual confounding—potentially arising from psychosocial factors, surgeon-level variation in surgical decision-making, and evolving tumor management guidelines—may still influence the observed associations. In addition, the SEER database lacks critical prognostic information, including Ki-67 status, lymphovascular invasion, details of neoadjuvant chemotherapy regimens, substantial missing data for reconstruction type, and the use of targeted and endocrine therapies, as well as patients' underlying medical conditions. Despite the inherent limitations of retrospective analyses and the ethical challenges associated with randomized controlled trials, the multivariable sensitivity analysis, PSM, and large sample size employed in this study provide robust insights into the long-term prognosis associated with surgical treatment strategies for breast cancer.

## Conclusions

In summary, this population-based analysis indicates that NSM with BR—but not TM with BR—is associated with improved OS and BCSS compared with TM alone among patients who received NAT. This association persisted across rigorous analytical methods and was particularly evident in younger patients with earlier-stage disease. These findings highlight NSM with BR as a surgically feasible option associated with favorable survival outcomes in selected patients in this observational cohort, warranting prospective validation.

## Funding

This study was supported by a grant from the Medical and Health Science and Technology Programme of Zhuji City, Zhejiang Province, China (2021YW009).

## CRediT authorship contribution statement

**ZhaoQi Qiu:** Writing – original draft, Formal analysis. **YuFeng Zhang:** Methodology, Formal analysis, Data curation. **Ting Shou:** Methodology, Formal analysis, Data curation. **YuRong Chen:** Writing – review & editing. **LieJiong Wang:** Methodology, Formal analysis, Data curation. **ZeMing Wang:** Supervision, Resources, Project administration, Conceptualization.

## Declaration of competing interest

The authors have declared no conflicts of interest.

## Data Availability

The data utilized and examined in this study are available from the corresponding author upon reasonable request.

## Appendix Supplementary materials

Image, application 1

Image, application 2

Image, application 3

Image, application 4
