# Supplementary figures and images for: Impact of breast reconstruction and different surgical approaches after neoadjuvant therapy on the long-term survival of breast cancer patients
Source: Transl Oncol. 2026 Mar 17;67:102737. doi: 10.1016/j.tranon.2026.102737 (PMC13011186; doi:10.1016/j.tranon.2026.102737)

variable

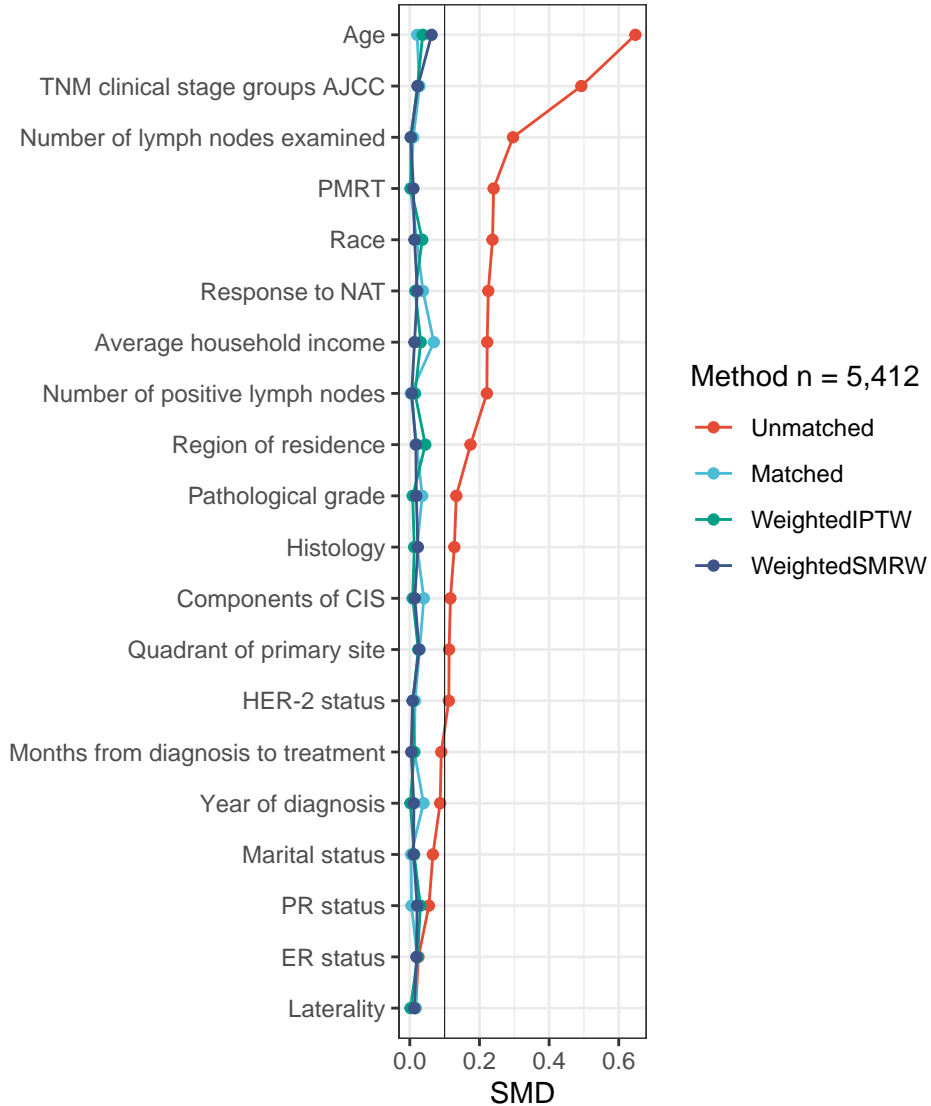

Supplement: Supplementary file 1 [file mmc1.pdf]

variable

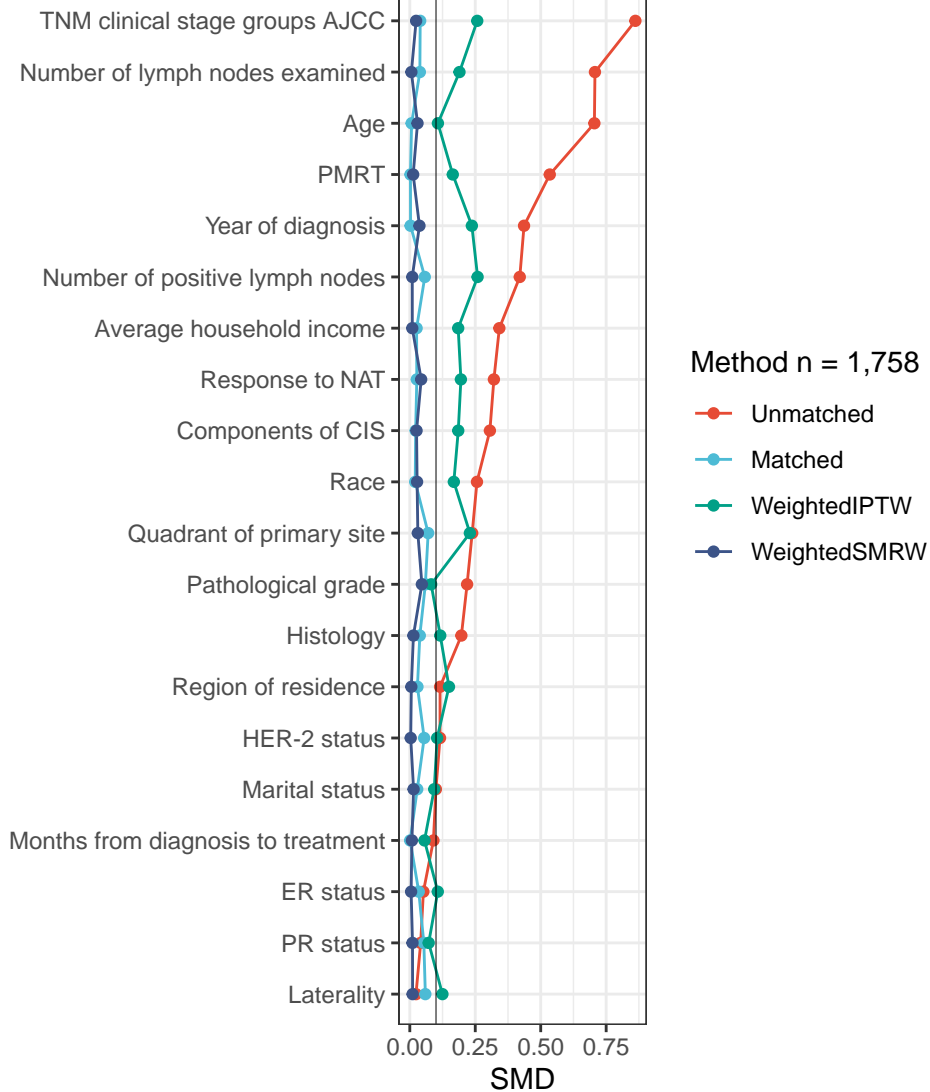

Supplement: Supplementary file 2 [file mmc2.pdf]
