# Supplementary material for: Impact of breast reconstruction and different surgical approaches after neoadjuvant therapy on the long-term survival of breast cancer patients
Source: Transl Oncol. 2026 Mar 17;67:102737. doi: 10.1016/j.tranon.2026.102737 (PMC13011186; doi:10.1016/j.tranon.2026.102737)
Supplement: Supplementary file 3 [file mmc3.docx]

| **Variables** | **Total**  **(n = 5412)** | **TM without BR**  **(n = 2706)** | **TM + BR**  **(n = 2706)** | **SMD** |
| --- | --- | --- | --- | --- |
| **Age, Mean ± SD** | 48.3 ± 11.0 | 48.2 ± 11.3 | 48.5 ± 10.6 | 0.021 |
| **Marital status, n (%)** |  |  |  | 0.004 |
| Unmarried | 1165 (21.5) | 584 (21.6) | 581 (21.5) |  |
| Married | 4072 (75.2) | 2034 (75.2) | 2038 (75.3) |  |
| Unknown | 175 (3.2) | 88 (3.3) | 87 (3.2) |  |
| **Race, n (%)** |  |  |  | 0.019 |
| Hispanic | 1134 (21.0) | 574 (21.2) | 560 (20.7) |  |
| Non-Hispanic White | 2867 (53.0) | 1423 (52.6) | 1444 (53.4) |  |
| Non-Hispanic Black | 777 (14.4) | 394 (14.6) | 383 (14.2) |  |
| Non-Hispanic Asian or Pacific Islander | 564 (10.4) | 280 (10.3) | 284 (10.5) |  |
| Unknown | 70 (1.3) | 35 (1.3) | 35 (1.3) |  |
| **Average household income, n (%)** |  |  |  | 0.069 |
| <$50,000 | 397 (7.3) | 212 (7.8) | 185 (6.8) |  |
| $50,000 - $75,000 | 2290 (42.3) | 1176 (43.5) | 1114 (41.2) |  |
| >$75,000 | 2725 (50.4) | 1318 (48.7) | 1407 (52.0) |  |
| **Region of residence** |  |  |  | 0.019 |
| Urban | 5033 (93.0) | 2511 (92.8) | 2522 (93.2) |  |
| Rural | 368 (6.8) | 190 (7.0) | 178 (6.6) |  |
| Unknown | 11 (0.2) | 5 (0.2) | 6 (0.2) |  |
| **Response to NAT, n (%)** |  |  |  | 0.038 |
| CR | 2062 (38.1) | 1020 (37.7) | 1042 (38.5) |  |
| PR | 1546 (28.6) | 768 (28.4) | 778 (28.8) |  |
| NR | 311 (5.7) | 150 (5.5) | 161 (5.9) |  |
| CR or PR | 1493 (27.6) | 768 (28.4) | 725 (26.8) |  |
| **Quadrant of primary site, n (%)** |  |  |  | 0.028 |
| Central quadrant | 220 (4.1) | 110 (4.1) | 110 (4.1) |  |
| Inner quadrant | 797 (14.7) | 406 (15.0) | 391 (14.4) |  |
| Outer quadrant | 2244 (41.5) | 1128 (41.7) | 1116 (41.2) |  |
| Axillary tail | 21 (0.4) | 9 (0.3) | 12 (0.4) |  |
| Overlapping lesion | 1235 (22.8) | 612 (22.6) | 623 (23.0) |  |
| Unknown quadrant | 895 (16.5) | 441 (16.3) | 454 (16.8) |  |
| **TNM clinical stage groups AJCC (8th), n (%)** |  |  |  | 0.028 |
| ⅠA | 604 (11.2) | 309 (11.4) | 295 (10.9) |  |
| ⅡA | 1678 (31.0) | 846 (31.3) | 832 (30.7) |  |
| ⅡB | 1241 (22.9) | 608 (22.5) | 633 (23.4) |  |
| ⅢA | 1041 (19.2) | 523 (19.3) | 518 (19.1) |  |
| ⅢB | 354 (6.5) | 175 (6.5) | 179 (6.6) |  |
| ⅢC | 494 (9.1) | 245 (9.1) | 249 (9.2) |  |
| **ER status, n (%)** |  |  |  | 0.021 |
| Positive | 3001 (55.5) | 1498 (55.4) | 1503 (55.5) |  |
| Negatives | 2383 (44.0) | 1196 (44.2) | 1187 (43.9) |  |
| Unknown | 28 (0.5) | 12 (0.4) | 16 (0.6) |  |
| **PR status, n (%)** |  |  |  | 0.005 |
| Positive | 2295 (42.4) | 1148 (42.4) | 1147 (42.4) |  |
| Negatives | 3086 (57.0) | 1543 (57.0) | 1543 (57.0) |  |
| Unknown | 31 (0.6) | 15 (0.6) | 16 (0.6) |  |
| **HER-2 status, n (%)** |  |  |  | 0.015 |
| Positive | 2135 (39.4) | 1059 (39.1) | 1076 (39.8) |  |
| Negatives | 3205 (59.2) | 1612 (59.6) | 1593 (58.9) |  |
| Unknown | 72 (1.3) | 35 (1.3) | 37 (1.4) |  |
| **Histology, n (%)** |  |  |  | 0.021 |
| IBC-NST | 5017 (92.7) | 2514 (92.9) | 2503 (92.5) |  |
| IBC-ST | 357 (6.6) | 175 (6.5) | 182 (6.7) |  |
| Rare and SGT | 38 (0.7) | 17 (0.6) | 21 (0.8) |  |
| **Laterality, n (%)** |  |  |  | 0.016 |
| Left side | 2772 (51.2) | 1375 (50.8) | 1397 (51.6) |  |
| Right side | 2640 (48.8) | 1331 (49.2) | 1309 (48.4) |  |
| **Number of lymph nodes examined, Median (IQR)** | 5.0 (2.0, 12.0) | 5.0 (2.0, 12.0) | 5.0 (2.0, 11.0) | 0.010 |
| **Number of positive lymph nodes, Median (IQR)** | 0.0 (0.0, 1.0) | 0.0 (0.0, 1.0) | 0.0 (0.0, 1.0) | 0.003 |
| **Months from diagnosis to treatment, Median (IQR)** | 1.0 (0.0, 1.0) | 1.0 (0.0, 1.0) | 1.0 (0.0, 1.0) | 0.013 |
| **Year of diagnosis, Median (IQR)** | 2018 (2015, 2019) | 2018 (2015, 2019) | 2017 (2015, 2019) | 0.039 |
| **PMRT, n (%)** |  |  |  | 0.001 |
| Yes | 2628 (48.6) | 1315 (48.6) | 1313 (48.5) |  |
| No | 2784 (51.4) | 1391 (51.4) | 1393 (51.5) |  |
| **Presence of CIS, n (%)** |  |  |  | 0.040 |
| No | 1060 (19.6) | 529 (19.5) | 531 (19.6) |  |
| Yes | 960 (17.7) | 460 (17.0) | 500 (18.5) |  |
| Unknown | 3392 (62.7) | 1717 (63.5) | 1675 (61.9) |  |
| **Pathological grade, n (%)** |  |  |  | 0.036 |
| Ⅰ | 208 (3.8) | 101 (3.7) | 107 (4.0) |  |
| Ⅱ | 1631 (30.1) | 827 (30.6) | 804 (29.7) |  |
| Ⅲ | 3236 (59.8) | 1601 (59.2) | 1635 (60.4) |  |
| Unknown | 337 ( 6.2) | 177 (6.5) | 160 (5.9) |  |

Appendix Table 5 Population characteristics of TM + BR vs. TM without BR after PSM

BR breast reconstruction; TM total mastectomy; NAT neoadjuvant therapy; CR complete response; PR partial response; NR no response; ER estrogen receptor; PR progesterone receptor; HER-2 human epidermal growth factor receptor 2; HR hormone receptor; IBC-NST invasive breast carcinoma of no special type; IBC-ST invasive breast carcinoma of special type; SGT salivary gland-type; PMRT postmastectomy radiotherapy; CIS carcinoma in situ; PSM propensity score matching
