# Supplementary material for: Impact of breast reconstruction and different surgical approaches after neoadjuvant therapy on the long-term survival of breast cancer patients
Source: Transl Oncol. 2026 Mar 17;67:102737. doi: 10.1016/j.tranon.2026.102737 (PMC13011186; doi:10.1016/j.tranon.2026.102737)
Supplement: Supplementary file 4 [file mmc4.docx]

| **Variables** | **Total**  **(n = 1758)** | **TM without BR**  **(n = 879)** | **NSM + BR**  **(n = 879)** | **SMD** |
| --- | --- | --- | --- | --- |
| **Age, Mean ± SD** | 46.4 ± 11.2 | 46.4 ± 11.7 | 46.5 ± 10.6 | 0.007 |
| **Marital status, n (%)** |  |  |  | 0.028 |
| Unmarried | 357 (20.3) | 174 (19.8) | 183 (20.8) |  |
| Married | 1357 (77.2) | 682 (77.6) | 675 (76.8) |  |
| Unknown | 44 (2.5) | 23 (2.6) | 21 (2.4) |  |
| **Race, n (%)** |  |  |  | 0.021 |
| Hispanic | 331 (18.8) | 165 (18.8) | 166 (18.9) |  |
| Non-Hispanic White | 1032 (58.7) | 519 (59.0) | 513 (58.4) |  |
| Non-Hispanic Black | 178 (10.1) | 87 (9.9) | 91 (10.4) |  |
| Non-Hispanic Asian or Pacific Islander | 200 (11.4) | 100 (11.4) | 100 (11.4) |  |
| Unknown | 17 (1.0) | 8 (0.9) | 9 (1.0) |  |
| **Average household income, n (%)** |  |  |  | 0.026 |
| <$50,000 | 74 (4.2) | 35 (4.0) | 39 (4.4) |  |
| $50,000 - $75,000 | 709 (40.3) | 358 (40.7) | 351 (39.9) |  |
| >$75,000 | 975 (55.5) | 486 (55.3) | 489 (55.6) |  |
| **Region of residence** |  |  |  | 0.029 |
| Urban | 1615 (91.9) | 811 (92.3) | 804 (91.5) |  |
| Rural | 143 (8.1) | 68 (7.7) | 75 (8.5) |  |
| **Response to NAT, n (%)** |  |  |  | 0.027 |
| CR | 791 (45.0) | 391 (44.5) | 400 (45.5) |  |
| PR | 481 (27.4) | 242 (27.5) | 239 (27.2) |  |
| NR | 88 (5.0) | 46 (5.2) | 42 (4.8) |  |
| CR or PR | 398 (22.6) | 200 (22.8) | 198 (22.5) |  |
| **Quadrant of primary site, n (%)** |  |  |  | 0.070 |
| Central quadrant | 53 (3.0) | 28 (3.2) | 25 (2.8) |  |
| Inner quadrant | 325 (18.5) | 160 (18.2) | 165 (18.8) |  |
| Outer quadrant | 741 (42.2) | 367 (41.8) | 374 (42.5) |  |
| Axillary tail | 11 (0.6) | 6 (0.7) | 5 (0.6) |  |
| Overlapping lesion | 375 (21.3) | 182 (20.7) | 193 (22.0) |  |
| Unknown quadrant | 253 (14.4) | 136 (15.5) | 117 (13.3) |  |
| **TNM clinical stage groups AJCC (8th), n (%)** |  |  |  | 0.040 |
| ⅠA | 304 (17.3) | 147 (16.7) | 157 (17.9) |  |
| ⅡA | 795 (45.2) | 397 (45.2) | 398 (45.3) |  |
| ⅡB | 377 (21.4) | 194 (22.1) | 183 (20.8) |  |
| ⅢA | 160 (9.1) | 80 (9.1) | 80 (9.1) |  |
| ⅢB | 39 (2.2) | 19 (2.2) | 20 (2.3) |  |
| ⅢC | 83 (4.7) | 42 (4.8) | 41 (4.7) |  |
| **ER status, n (%)** |  |  |  | 0.034 |
| Positive | 955 (54.3) | 476 (54.2) | 479 (54.5) |  |
| Negatives | 795 (45.2) | 398 (45.3) | 397 (45.2) |  |
| Unknown | 8 (0.5) | 5 (0.6) | 3 (0.3) |  |
| **PR status, n (%)** |  |  |  | 0.054 |
| Positive | 729 (41.5) | 374 (42.5) | 355 (40.4) |  |
| Negatives | 1017 (57.8) | 498 (56.7) | 519 (59) |  |
| Unknown | 12 (0.7) | 7 (0.8) | 5 (0.6) |  |
| **HER-2 status, n (%)** |  |  |  | 0.055 |
| Positive | 678 (38.6) | 331 (37.7) | 347 (39.5) |  |
| Negatives | 1065 (60.6) | 542 (61.7) | 523 (59.5) |  |
| Unknown | 15 (0.9) | 6 (0.7) | 9 (1.0) |  |
| **Histology, n (%)** |  |  |  | 0.038 |
| IBC-NST | 1661 (94.5) | 831 (94.5) | 830 (94.4) |  |
| IBC-ST | 76 (4.3) | 36 (4.1) | 40 (4.6) |  |
| Rare and SGT | 21 (1.2) | 12 (1.4) | 9 (1.0) |  |
| **Laterality, n (%)** |  |  |  | 0.059 |
| Left side | 918 (52.2) | 472 (53.7) | 446 (50.7) |  |
| Right side | 840 (47.8) | 407 (46.3) | 433 (49.3) |  |
| **Number of lymph nodes examined, Median (IQR)** | 3.0 (2.0, 6.0) | 3.0 (2.0, 6.0) | 3.0 (2.0, 6.0) | 0.038 |
| **Number of positive lymph nodes, Median (IQR)** | 0.0 (0.0, 0.0) | 0.0 (0.0, 0.0) | 0.0 (0.0, 0.0) | 0.057 |
| **Months from diagnosis to treatment, Median (IQR)** | 1.0 (1.0, 1.0) | 1.0 (1.0, 1.0) | 1.0 (1.0, 1.0) | 0.001 |
| **Year of diagnosis, Median (IQR)** | 2018 (2016, 2019) | 2018 (2016, 2020) | 2018 (2016, 2019) | 0.003 |
| **PMRT, n (%)** |  |  |  | 0.002 |
| Yes | 565 (32.1) | 283 (32.2) | 282 (32.1) |  |
| No | 1193 (67.9) | 596 (67.8) | 597 (67.9) |  |
| **Presence of CIS, n (%)** |  |  |  | 0.022 |
| No | 240 (13.7) | 117 (13.3) | 123 (14.0) |  |
| Yes | 194 (11.0) | 96 (10.9) | 98 (11.1) |  |
| Unknown | 1324 (75.3) | 666 (75.8) | 658 (74.9) |  |
| **Pathological grade, n (%)** |  |  |  | 0.059 |
| Ⅰ | 69 (3.9) | 39 (4.4) | 30 (3.4) |  |
| Ⅱ | 508 (28.9) | 254 (28.9) | 254 (28.9) |  |
| Ⅲ | 1109 (63.1) | 548 (62.3) | 561 (63.8) |  |
| Unknown | 72 (4.1) | 38 (4.3) | 34 (3.9) |  |

Appendix Table 6 Population characteristics of NSM + BR vs. TM without BR after PSM

BR breast reconstruction; TM total mastectomy; NSM nipple-sparing mastectomy; NAT neoadjuvant therapy; CR complete response; PR partial response; NR no response; ER estrogen receptor; PR progesterone receptor; HER-2 human epidermal growth factor receptor 2; HR hormone receptor; IBC-NST invasive breast carcinoma of no special type; IBC-ST invasive breast carcinoma of special type; SGT salivary gland-type; PMRT postmastectomy radiotherapy; CIS carcinoma in situ; PSM propensity score matching
